# Simulating Metabolism with Statistical Thermodynamics

**DOI:** 10.1371/journal.pone.0103582

**Published:** 2014-08-04

**Authors:** William R. Cannon

**Affiliations:** Computational Biology and Bioinformatics Group, Fundamental and Computational Sciences Directorate, Pacific Northwest National Laboratory, Richland, Washington, United States of America; Universitat Pompeu Fabra, SPAIN

## Abstract

New methods are needed for large scale modeling of metabolism that predict metabolite levels and characterize the thermodynamics of individual reactions and pathways. Current approaches use either kinetic simulations, which are difficult to extend to large networks of reactions because of the need for rate constants, or flux-based methods, which have a large number of feasible solutions because they are unconstrained by the law of mass action. This report presents an alternative modeling approach based on statistical thermodynamics. The principles of this approach are demonstrated using a simple set of coupled reactions, and then the system is characterized with respect to the changes in energy, entropy, free energy, and entropy production. Finally, the physical and biochemical insights that this approach can provide for metabolism are demonstrated by application to the tricarboxylic acid (TCA) cycle of Escherichia coli. The reaction and pathway thermodynamics are evaluated and predictions are made regarding changes in concentration of TCA cycle intermediates due to 10- and 100-fold changes in the ratio of NAD^+^:NADH concentrations. Finally, the assumptions and caveats regarding the use of statistical thermodynamics to model non-equilibrium reactions are discussed.

## Introduction

Ideally, models of metabolism should predict metabolite levels, characterize the thermodynamic requirements of pathways and processes, be testable with experimental data, and provide insight into the principles of cellular function and self-organization. Simulations based on the law of mass action, such as kinetic simulations, can in principle meet these requirements. However, these simulations require knowledge of the thousands of rate constants involved in the reactions. The measurement of rate constants is very labor intensive, and hence rate constants for most enzymatic reactions are not available. Moreover, the same *prima facie* enzymes (ortholog) from different species, or even different strains, have differing rate constants. For example, for dihydrofolate reductase, the turnover rates for the substrate 7,8-dihydrofolate measured *in vitro* vary five orders of magnitude across species – from 284 s^−1^ to less than 1 s^−1^ [1]. If one were to model the metabolism of an organism using kinetic simulations, the rate constants for each enzyme would first need to be measured.

Currently, flux-based approaches are the methods of choice for modeling metabolism because they do not require the use of rate constants. Instead, flux-based approaches are based on fitting reaction flux values to an experimentally measured growth rate. However, this computational convenience also limits the predictive power of the methods, in that the prediction of metabolite levels from flux values [2]–[4] relies on the assumption of reversibility for non-equilibrium reactions [5], [6]. Consequently, predicted levels of metabolites may have large uncertainties, especially when the range of calculated fluxes consistent with the steady state assumption can span many orders of magnitude [7]. Moreover, flux-based methods do not provide any information on energy requirements of pathways, sets of pathways, or organisms in a community without likewise making assumptions about the reversibility of non-equilibrium reactions [8], [9].

An alternative to both these approaches is to model metabolism using simulations of states rather than simulations of reactions. State-based simulations were in fact the first computer simulations ever performed and were reported in the classic Metropolis Monte Carlo paper, *Equation of State Calculations by Fast Computing Machines*, which came out of the Manhattan project [10]. Similar time-independent, statistical mechanical methods are now widely used in adsorption physics [11], quantum Monte Carlo simulations [12], protein engineering [13], drug design [14] and elsewhere. In the context of metabolic modeling, the state consists of the set of concentrations of all metabolites. A change of state occurs when the concentrations of the metabolites change due to a reaction. The change of state is evaluated using state function such as the Gibbs energy, the isothermal-isobaric free energy, or any other appropriate state function. Therein lies the advantage of simulations that model states rather than time-dependent reactions – the parameters needed to model states (standard free energies of reaction) are much easier to determine than the parameters needed to model reactions (rate constants). An assumption used in this study is that each reaction occurs with a frequency proportional to the thermodynamic driving force on the reaction, although this assumption can be alleviated by including activities in the model. The disadvantage is that the specific time-dependence of each reaction is lost, which has some consequences as discussed below. The simulations can be carried out stochastically or deterministically, and equilibrium as well as nonequilibrium processes can be modeled. The usual caveat for state simulations regarding dynamic bottlenecks in phase space apply [15], and are discussed below.

As biology emerges as a physical science, researchers will likely need different modeling approaches – kinetic, flux-based, or statistical thermodynamic – based on the details of the question being asked. In computational chemistry, for example, many different models are employed depending on the research question, ranging from electronic structure calculations with electron correlation, to hybrid QM/MD, to molecular mechanics models. The approach outlined in the paper can provide a detailed model of metabolism that provides in-depth information, but not all questions may require this level of information.

Here, the basic aspects of the statistical thermodynamics background needed for simulating metabolic systems are presented. The methods section does require some mathematical background in multinomial statistics, however this background is not necessary to understand the application presented in the results section. The application is that of the tricarboxylic acid cycle from *Escherichia coli*, for which the free energy, energy and entropy profiles are determined as well as predictions of metabolite concentrations. However, the point of this report is not to model a particular process in high fidelity, but rather to demonstrate the principles of applying statistical thermodynamics to metabolic reaction networks. Finally, this report concludes with a discussion of the advantages and limitations of using state-based simulations to model metabolism.

## Methods

### Theory - Statistical thermodynamics of coupled reactions

This section provides the basic statistical thermodynamic background that is needed to implement and characterize simulations of coupled reactions based on modeling states. Consider the reaction,
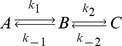
(Scheme 1)


This set of reactions is comparable to the conversion of citrate to isocitrate via aconitate in central metabolism in which the waters involved in the reactions are modeled implicitly. The number *m* of molecular species is three (*A*, *B* and *C*), the number of particles of type *j is n_j_*, and there are 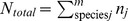 total particles in the system. For classical systems, the distribution of states are rooted in Boltzmann statistics in which the particles are assumed to be distinguishable. The partition function for the system is 
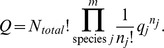



The *q_j_* are the molecular partition functions for species *j*, and 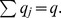 Conceptually, *q* is not only the sum of the molecular partition functions, but it is also the molecular partition function of the hypothetical boltzon particle that can be in one of *m* states with corresponding energy levels 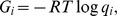 *i* = 1…*m*. When corrected for indistinguishability (corrected Boltzmann statistics), the partition function for the system is given by [16], [17],



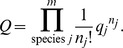



The issue whether to use Boltzmann or corrected Boltzmann statistics depends on whether the particles are distinguishable or not. For example, when considering a high density of particles at low temperature such that the N-body Schrodinger equation can't be separated, corrected Boltzmann statistics are appropriate. However, the interest here is in considering particles that exist as distinguishable chemical species. As a result, in the rest of the discussion and for demonstration purposes Boltzmann statistics will be used.

In statistical thermodynamics the free energy of the system is,
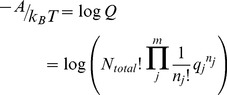



In comparison, the multinomial probability mass density for a set of independent and distinguishable objects is,
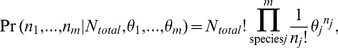
(1)where 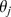 is the probability of an object being of type *j*. Identifying objects with chemical species, and taking the species probability as the Boltzmann probability, 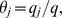 the free energy can be expressed in terms of the probability mass density [18],



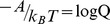


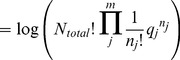
(2)

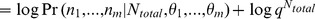
(3)


The difference between the multinomial probability mass function 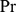 and the system partition function 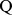 is 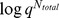
– the log of a multinomial expansion of *q*. The multinomial expansion is simply the number of configurations that the system can obtain – the extent of state space,
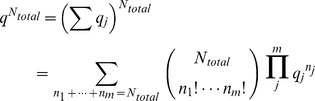



In contrast, the multinomial expansion used in the probability mass function is,
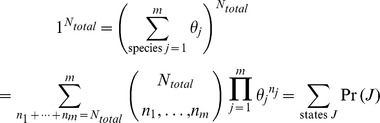
where 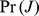 is shorthand for 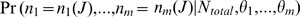. As long as *N_total_* is constant, the probability of state *J* is given by the multinomial probability mass function.

**Figure 1:**
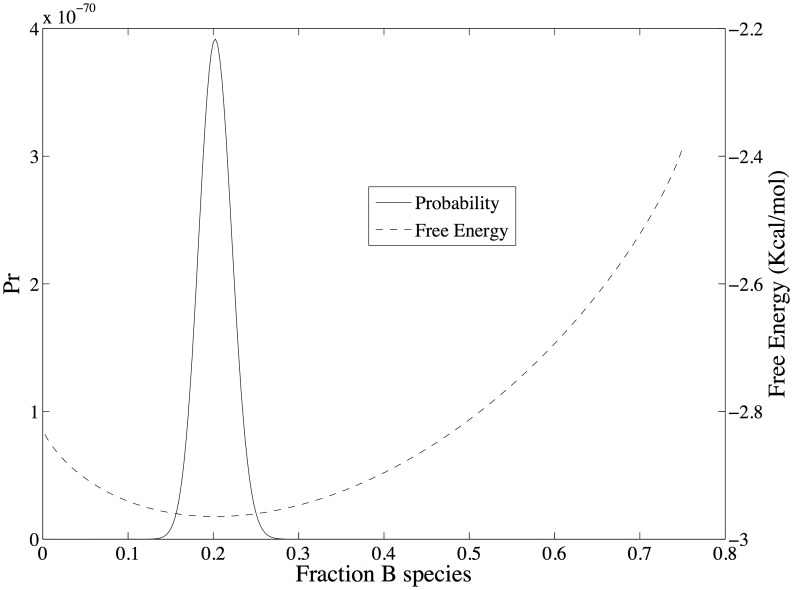
Probability densities mapped on to the total free energy surface (Equation 3) for reaction scheme 1. The number of particles for species A is held fixed and only species B and C subject to the constraint *n_A_* + *n_B_* + *n_C_* = *N_total_*. Values for *n_A_*, *N_total_*, and the free energy of formation of each species and other parameters are given in Table 1, column 1.

To demonstrate the relationship between free energy and probability for Scheme 1, the probabilities 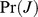 as a function of the fraction of *B* species are mapped on to the total free energy surface (Equation 3) in Figure 1. In this example, the number of particles for species *A* is held fixed and only species *B* and *C* vary, subject to the constraint *n_A_* + *n_B_* + *n_C_* = *N_total_*. Values for *n_A_*, *N_total_*, and the free energy of formation of each species and other parameters are given in Table 1, column 1. The probability density is shown as a function of the fraction of *B* species, 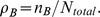 The probability density is at a maximum of 1.63×10^−2^ at 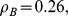 exactly where the free energy is at a minimum.

**Table 1:** Parameters used for modeling the reactions shown in Scheme 1 for Figures 1–6.

Parameter	Figures 1–4	Figures 5–6
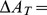	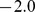	
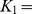	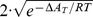	
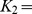	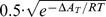	
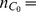	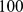	
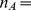	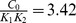	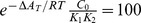
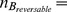	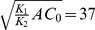	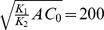
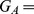	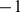	
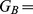	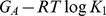	
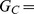	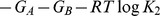	
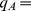	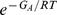	
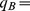	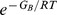	
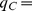	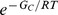	
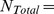	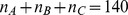	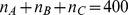
*n_B_* =	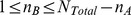	
*n_C_* =	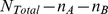	

### Configurational Energy and Entropy

The free energy above is an extensive function of the system because of the dependence on the total number of particles. The free energy per molecule or mole is often more useful for comparative purposes. When normalized by the total number of particles, the resulting scaled free energy 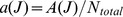 is,
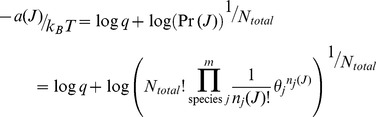
(4)


The second term is the log of the geometric mean probability per particle. The usual thermodynamic formulation of the entropy and energy can be recovered from Equation 4 using Sterling's approximation and separating the combinatorial terms from the species probability 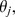


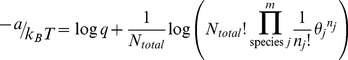


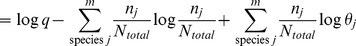
(5)


Writing the Boltzmann probabilities in Equation 5 in their explicit form, and recognizing that 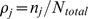 is the fraction of the total population that exists as species *j*, 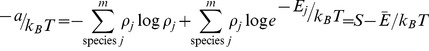
(6)


In Equation 6, the first term is the configurational entropy [16]. The density 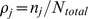 is a probability of the uniform probability distribution, thus the configurational entropy is an information entropy of the uniform probability distribution, not the Boltzmann distribution. The second term in Equations 6 is simply the average energy at the location {*n_A_*, *n_B_*, *n_C_*} in state space.

**Figure 2:**
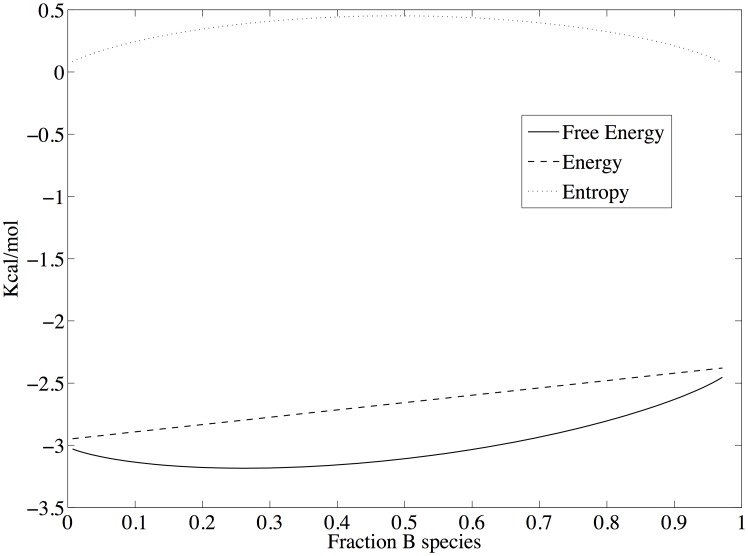
Energy, entropy and free energy of reaction scheme 1 as a function of the fraction of species B. The reaction conditions are the same as in Figure 1, and are given in Table 1, column 1.

Figure 2 shows the total energy, entropy and free energy of Scheme 1 as a function of the fraction of species *B*. The reaction conditions are the same as those used for Figure 1. Since only species *B* and *C* can vary in this example and *C* has a lower free energy of formation than *B*, the total energy of the system is a linearly decreasing function of the fraction of *B* species. In contrast the entropy is a convex function of the fraction of *B* species with a maximum when there is an equal amount of each species, as one would expect from a uniform probability distribution. Consequently, the free energy minimum is a tradeoff between an entropy that is maximized when particles are uniformly distributed and an energy that is minimized with an increasing number of the lowest energy species, *C*. The free energy minimum corresponds with the maximum probability density (Figure 1). g

### Changes of State

Next, consider changes in the counts of individual particles, but such that the total number of particles is constant. The system may be considered to be closed and the changes in concentrations are due to fluctuations, or alternatively, the system may be open such that the number of particles coming into the system is equal to the number of particles going out, and the changes in the relative concentrations are due to steady state processes.

The difference in free energy between two states in which *N_total_* is constant is,
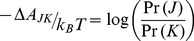
(7)

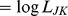
where 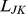 is the likelihood ratio of the probability mass functions, Pr(*J*)/Pr(*K*). If one allows changes between states 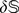 to occur only through a single firing of individual reactions, a Markov chain in state space exists in which the probability of state *J* at step *i* is represented by,



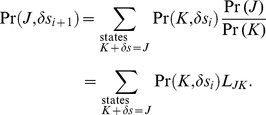
(8)To be clear, Equation 8 does not imply a relationship involving time; it is simply a statistical relationship between states without regard for time dependence. This should be clear from the functional form of Equation 8, which is quite different from the functional form for a time-dependent rate equation,
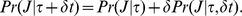



Because of differences in the time dependence of reactions, however, an actual system may not exhibit the ideal behavior implied by Equation 8. If used for modeling reactions, Equation 8 contains an assumption that at each possible change of state, the choice is based on reversible thermodynamic likelihoods. That is, for any change of state due to the firing of a single reaction, the change of state can be modeled using the Boltzmann distribution. This is a “local equilibrium” assumption and is equivalent to assuming that the frequency of each reaction is proportional to the thermodynamic driving force of the reaction. A similar assumption is used in transition state theory [19], yet here the assumption is not as severe; unlike transition state species, chemical intermediates in a reaction pathway are stable compounds that can be isolated.

### Changes in Thermodynamic Functions

Regarding changes in probability density due to changes in state, the difference in mean free energy per particle between states *J* and *K* when the total number of particles does not change but the count of any species can vary is given by,
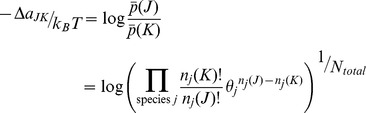



The difference in mean free energy per particle between states *J* and *K* is the mean difference in the probability mass density for each state. When the difference is large, the probability of one state is greater than probability of the second state. The sign of the difference indicates which state is more probable.

The differences in entropy and energy between states on a per particle basis are again obtained by separating the terms for the individual species probability and combinatorics, 
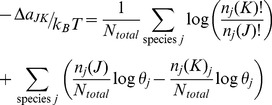
(9)


Using Sterlings approximation again, the first summation can be rewritten as,
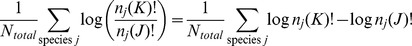
(10)

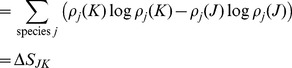
(11)


The last equality again is the entropy difference in state space according to the uniform probability distribution.

**Figure 3:**
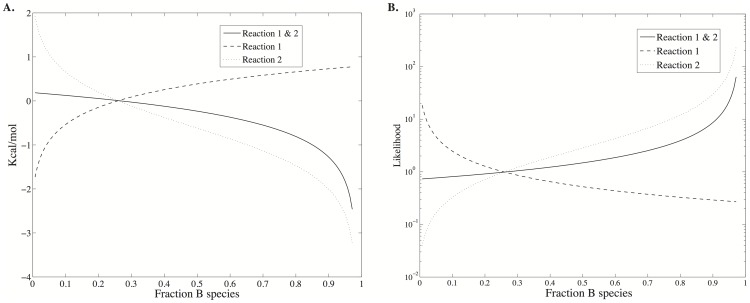
The change in free energy and likelihood across the reactions shown in Scheme 1. **A.** The equilibrium position is where the total free energy change (solid line) crosses the ordinate at 0.0 Kcal Kcal/mol and the abscissa at 0.26 *n_B_*/*N_total_*. In accordance with detailed balance, the free energy change for each reaction is also 0 Kcal Kcal/mol. **B.** The combined likelihood for the reactions (solid line) is 1.0 at equilibrium, just as the likelihoods for each reaction individually.

The second summation in Equation 9 is equal to the difference in the average energy between the states, 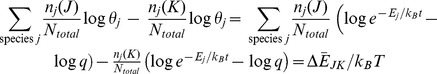


Using the parameters in Table 1, column 2, the change in free energy for traversing the reactions in Scheme 1 is shown in Figure 3A. The equilibrium location of the system occurs at the minimum in the total free energy (Figure 2) at *r_B_* = 0.26. Shown in Figure 3B are the likelihoods of a change of state according to Equation 7 for reactions 1 and 2 individually as well as for the system of coupled reactions. At equilibrium, the likelihoods of a change of state are equal for the forward and reverse steps of each reaction, in accordance with detailed balance.

**Figure 4:**
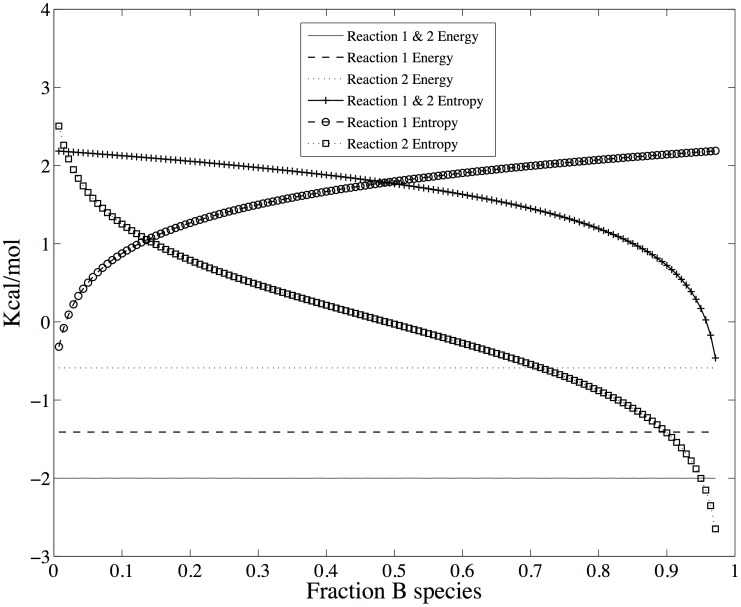
Changes in energy and entropy across the reactions shown in Scheme 1. In contrast to the total energy shown in Figure 2, the change in energy is independent of the starting state of the system because the stoichiometry change is always the same. The change in entropy, however, is not constant.

As one moves away from equilibrium, the likelihood of a change of state is in accordance with Le Chatelier's principle to restore equilibrium. Shown in Figure 4 are the changes in energy and entropy for the reactions of Scheme 1 under the conditions of Table 1. Regardless of the initial state of the system (indicated by 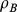 in the figures), the energy change for each reaction is dictated by the stoichiometry of the reaction and always involves a decrease in one *A* particle and increase of one *B* particle for reaction 1, and a decrease of one *B* particle and an increase in one *C* particle for reaction 2. As a result, the energy change for each reaction, and hence for both reactions together, does not depend on the state of the system. The driving force to restore equilibrium is entirely entropic – due to the greater number of ways of combinatorially arranging the particles - as indicated by the variation in entropy with 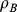 shown in Figure 4.

At the equilibrium position of 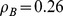, 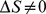 for a change of state of either the system or for each reaction individually. However, this does not violate the principle that the change in entropy for a dynamic system at equilibrium is zero. Rather, the rate of production of configurational entropy changes due to the reactions, 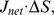 (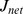 is the net flux of a reaction) is zero because the net flux is zero, not because 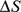 is zero. (*N.b.*, the concept of entropy production is generally discussed in regard to the entropy of the state, not the configurational entropy – see below.)

Just away from equilibrium, the production rate for the configurational entropy may not be minimal, however, since 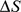 may be large. In fact, since the configurational entropy is based on the uniform distribution, 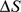 approaches zero when the initial reactants and final products are equally distributed. Importantly, the configurational entropy is a measure of the uniform probability distribution, which is not the natural probability distribution of the species. Hence, the configurational entropy may not be at a maximum when the system is fully relaxed (equilibrium).

### Equilibrium and Non-Equilibrium Steady States

The example discussed above contains the equilibrium steady state. At equilibrium the thermodynamic likelihood (Equation 7) of a change of state from the free energy minimum state to a state in the neighborhood of the free energy minimum state is approximately 1.0. If the free energy minimum state is 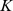 and those in the neighborhood of the minimum state are 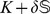 then, 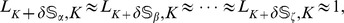



Here, *K* is the state of the system before a reaction and 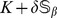 is the state of the system after reaction 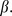 In any real system, the values of the likelihood will fluctuate around the average value of 1.0.

Using this same approach, a thermodynamically stable, non-equilibrium steady state can also be characterized. As in the equilibrium case, thermodynamic stability occurs when each reaction in a pathway is equally likely, but the likelihood is not necessarily 1.0. Using the likelihood of a change of state due to a reaction *i* for an open system, 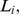 the likelihood of each reaction of the system can be any value such that,
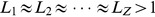
(12)where *Z* is the number of reactions in the pathway, or in terms of equilibrium constants *K_eq_* and reaction quotients *Q_i_*,



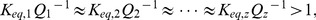
(13)


Equation 12 can be expressed as a maximum entropy requirement in which the state entropy 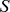 in a state space neighborhood 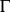 measures the probability density of states reachable from an initial state 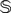 due to a series of *Z* reactions involving a change of state 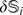,
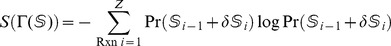
(14)


The state entropy increases as the system stabilizes, and reaches a maximum at equilibrium since equilibrium requires that each respective reaction is equally likely. In a non-equilibrium system, the neighborhood 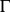 is a reaction path and Equation 14 is the path entropy described by Dewar, from which the fluctuation theorem, the selection principle of maximum entropy production, and self-organized criticality can be derived [20].

For reference, the state entropy at equilibrium can be compared to the state entropy in absence of any reactions or ability of the system to change states. For instance, a convenient reference state would have all particles in the system existing as the initial reactants, which for Scheme 1 would be such that 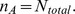 If the chemical species *A* has the highest free energy of formation (hence the lowest Boltzmann probability), then this state (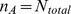) has the lowest probability density and state entropy. Here the state entropy of this reference state is indicated by 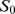 to indicate that it is the state entropy in the neighborhood of complete order. The change in entropy from this reference state to any other state is then 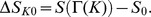 Since the state entropy is a probabilistic measure of the free energy, 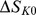 is a measure of the free energy dissipation due to the presence of the reactions, which for biological systems would measure the in energy dissipation due to biological processes. Using this definition of state entropy, the entropy production rate can be characterized as,
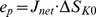



At equilibrium, the entropy production rate for the state entropy defined above is zero since the net flux 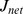 is zero. In addition, since 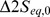 is the maximum of 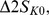 the change in the state entropy due to any reaction around equilibrium is 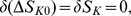 on average.

**Figure 5:**
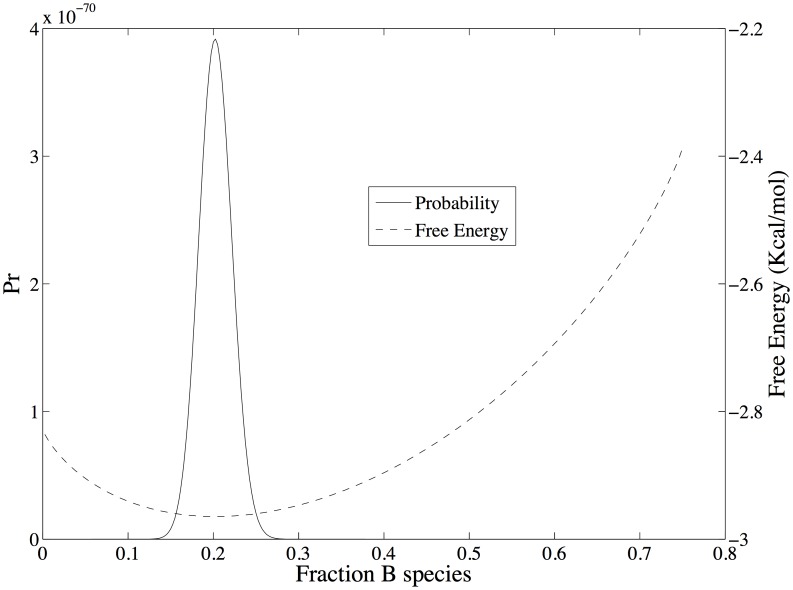
The state probability density and total free energy for the coupled reactions of scheme 1 that includes a non-equilibrium thermodynamic stable state. Parameters are given in Table 1, column 2. In this case, the equilibrium/most probable state on the abscissa at 0.20 *n_B_*/*N_total_*. The nonequilibrium steady state is located on the abscissa at 0.50 *n_B_*/*N_total_*, and is stable only because of the applied driving force of -2 Kcal Kcal/mol (Figure 7).

**Figure 6:**
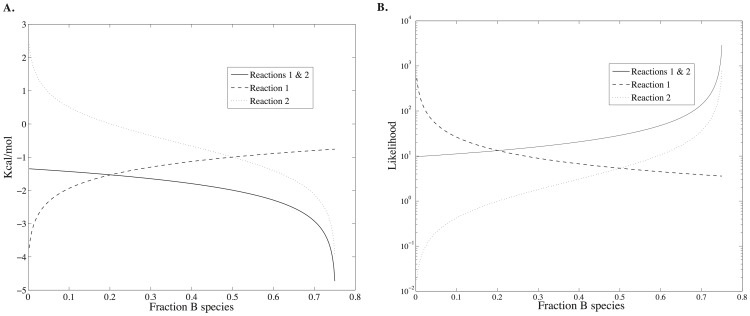
Shows the change in free energy (A) and log likelihood (B) for the coupled reactions of scheme 1. The non-equilibrium thermodynamic stable state occurs at *ρ_B_* = 0.5.

A non-equilibrium, thermodynamically stable state occurs at the point at which Equation 12 is satisfied, or when the state entropy (Equation 14) no longer increases. The state probability density and total free energy for the coupled reactions of Scheme 1 that includes a non-equilibrium thermodynamic stable state is shown in Figure 5. The stable state is maintained by constraining the population of only species *A* in Scheme 1. The parameters used here are similar to those used in Figures 1–4 and are listed in Table 1, column 3; only the count of species *A* is different, which has been adjusted to provide an overall driving force of -2.0 Kcal/mol. The most probable state, which is a function of the total free energy of the system according to Equation 3, is now at 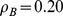. In this state, the species are distributed according to the Boltzmann distribution with 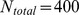 particles with the constraint that *n_A_* = 100 particles. Because of the latter constraint, the probability distribution is optimized only with respect to reaction 2 – only species *B* and *C* can vary. Consequently, as shown in Figures 6A and 6B, the likelihood ratio of the forward to reverse reaction for reaction 2 is 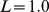 at 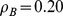, and the free energy change, which is the log of the likelihood ratio, is 0.0. However, since the likelihood of reaction 1 is much greater than the likelihood of reaction 2 when *n_A_* is constrained to 100 particles, the state at 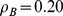 is not thermodynamically stable with respect to the full state space - it is only the most probable state with respect to counts of species *B* and *C*. When *n_A_* is unconstrained and *N_total_* = 400, the most probable state (and global free energy minimum) is at *n_A_* = 10, *n_B_* = 105, and *n_C_* = 285 (not shown).

The stable state at which the likelihood of each reaction is equal occurs when the count of species *C* is also fixed such that 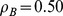, as shown in Figure 6 A and B. At this state, the log likelihood of each individual reaction is 0.72 (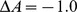
Kcal/mol), corresponding to a log likelihood ratio of 1.44 for the coupled reactions (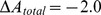
Kcal/mol), as shown in Figure 6. As in the equilibrium case shown in Figure 4, the energy change for each reaction, and hence for both reactions together, does not depend on the state of the system since the energy change always involves a decrease in one *A* particle and increase of one *B* particle for reaction 1, and a decrease of one *B* particle and an increase in one *C* particle for reaction 2. What is different is that now the free energy change for traversing the reactions is not zero but matches the change in energy. Since the change in free energy matches the change in energy, the change in configurational entropy for the reactions is zero. This is the nature of a stable state by definition – there are no changes in the counts/concentrations of the chemical species. As mentioned above, the configurational entropy is based on the uniform probability distribution and hence is at a local or global maximum when the counts/concentrations of initial reactants and final products are equal. Consequently, the change in configurational entropy 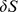 with respect to small changes in the distribution is zero - the change in the configurational entropy due to reaction 1 is exactly balanced by the change in configurational entropy due to reaction 2 – a minimum entropy production principle. More generally, for any coupled reactions in a thermodynamically stable state, there is no change in the configurational entropy due to the stable state. This is a principle of minimum entropy change for a stable state.

Summarizing, for a thermodynamic stable state, the rate of production of changes in configurational entropy, 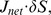 is zero just as in the equilibrium case but for a different reason: now the change in configurational entropy is zero but the net flux 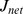 is not. Consequently, the rate of production of changes in configurational entropy is at a minimum at both the equilibrium and non-equilibrium stable states, but for different reasons.

One final note regarding the configurational entropy. Since the configurational entropy is based on the uniform probability distribution and not the natural Boltzmann probability distribution, a relatively high value of the configurational entropy may indicate significant order in a system, not disorder or variation.

In contrast, a high value of the state entropy indicates that the system is distributed more or less in accord with its natural distribution. The state entropy production rate for a non-equilibrium stable state *K,* 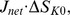 is greater than zero since both the state entropy 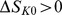 (by definition above) and 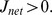 Although, 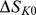
may be at a local maximum at a non-equilibrium stable state, it is nevertheless less than 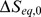, indicating that the system is ordered relative to equilibrium.

### Work done in establishing the stable state

In order to maintain the stable state, work must be done by an external force against the tendency of the system to return to equilibrium. For Scheme 1, the work consists of moving the system from the (global) minimum free energy configuration to a configuration in which the work required to create one *C* species from *A* is −2.0 Kcal/mol. In this case, the work done to maintain the steady state is 0.38 Kcal/mol. The general principle here is that large thermodynamic driving forces give rise to otherwise improbable configurations. These configurations are dissipative structures, and self-organization is reflected in the configurational entropy. This may seem counter-intuitive that, for instance, a maximization of entropy may be a sign of organization, but one must keep in mind that the natural distribution is the Boltzmann distribution and the configurational entropy is based on the uniform distribution. (In the trivial case when the Boltzmann probability of each species is equal, the uniform probability distribution also becomes a natural distribution for these species.)

### Comparison of stable state and steady state requirements

The thermodynamic stability requirement of Equation 12 can be contrasted with the steady state requirement in which the net flux of each reaction must be the same,
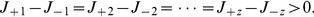
(15)


This relationship, in fact, also holds for thermodynamically stable states as a consequence of Equation 12. Although it is tempting to infer additional relationships between flux and free energy, the issue is complicated by the reaction statistics. The free energy change pertains to a *single* change of state, and in the context of this report the change of state due to a single reaction. In contrast, the flux is a statistic of measuring *multiple* changes of state, and is an emergent property of the system.

### Open system with varying number of particles

For a non-equilibrium steady state system in which the total number of particles can vary except the boundary particles – which are the sources and sinks of material - the thermodynamic probability of a state 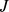 is given by a probability density similar to the multinomial Boltzmann distribution used in Equations 1–3 but with the counts of the boundary species fixed and the multinomial expansion of the molecular partition function of the hypothetical boltzon (*q*) explicitly represented in the distribution to account for normalization of the probability density,
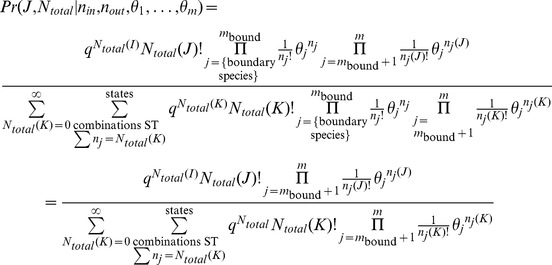
(16)


Analogous to Equation 7 for a system with a constant number of total particles, the odds of a change of state for an open system is given by,
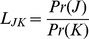



The ratio of the open system probabilities is the likelihood ratio, 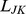, and the free energy change for a change of state is just the log likelihood, 
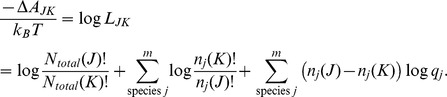



In discussing an open system, it is more convenient to formulate the free energy using molecular partition functions, 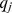, rather than the corresponding Boltzmann probabilities, 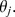 Expressed in terms of the mean free energy per mole and combining the factorial terms,
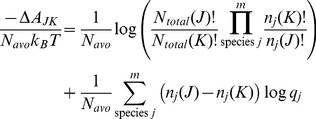
(17)

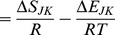
where 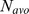 is Avogadro's number and 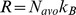 is the gas constant. Of course, 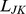 can also be obtained as the likelihood ratio of the concentrations of the reaction products and reactants at reference values to those at non-reference values - usual product of the equilibrium constant and the reciprocal of the reaction quotient, 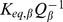 for a reaction 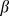 that involves a change of state from *K* to *J*.

Due to constraints at the boundary of the system, the probability of a state determined from Equation 16 is not the same as the equilibrium probability unless the boundary species are also at equilibrium. When the state space is sampled according to the Boltzmann probabilities the average number of particles is given by,
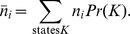



However, the actual time-dependence of the reactions may create bottlenecks in state space such that the sampling of state space does not follow the Boltzmann probabilities. For instance, in Scheme 1 the product *B* of the first reaction may be removed quickly by the second reaction before it has a chance to become distributed according to the Boltzmann probabilities. For instance, both organic and biochemical systems are known to use high-energy reaction products as intermediates in otherwise thermodynamically favorable pathways. This is why one must be careful in drawing conclusions about metabolite concentrations, flux values [3], [4] or reaction energetics [8], [9] when using the assumption of detailed balance to analyze non-equilibrium reactions [5], [6]. The time-dependent state probabilities can be sensitive functions of the transition rates between states. In such cases the states are sampled such that the average number of particles is given by, 
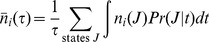
or using discrete time steps 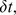




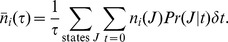



The observed sampling may not result in a maximum likelihood when the underlying distribution is assumed to be a Boltzmann distribution.

Below, the symbol _*_ is used to indicate the average value of a property when the sampling of reactions follows a thermodynamic likelihood distribution - the reaction with the most favorable free energy change is more likely than a reaction with a less favorable free energy changes as indicated by Equation 17. That is, rather than a reaction being sampled according to a specific time-dependent likelihood, the likelihood of an individual reaction follows a Boltzmann likelihood. This does not necessarily imply equilibrium, however, because as mentioned above, the boundary conditions of the system may not be at equilibrium. Using this approach, we relate the concepts of energy and entropy dissipation discussed by Ge and Qian [21] to the framework presented here.

If the expected number of *A* particles in the most probable state is 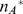, then the mean free energy per particle difference between the most probable state and state *K* is,
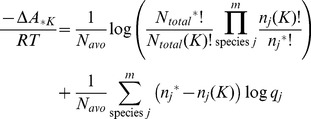



The first summation is the instantaneous configurational entropy difference due to a fluctuation, while the second summation is the instantaneous heat production/dissipation due to a fluctuation. The total free energy of the system can be rewritten as the sum of the free energy of the thermodynamically most probable state and the difference in free energy between the most probable state and any other state due to, for instance, a fluctuation,
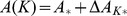
or,



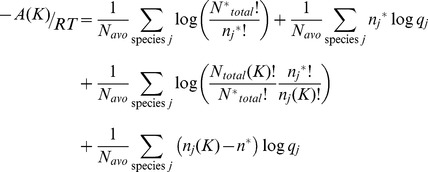
(18)The first two summations are the configurational entropy production and heat production due to non-equilibrium stable-state conditions, respectively, while the last two summations are the configurational entropy and heat dissipation/absorption due to a spontaneous fluctuation – a single change of state - away from the thermodynamically most stable state.

### Excess Free Energy, Entropy and Heat

As mentioned above, it may be that some states are highly probable when reactions are sampled based on Boltzmann likelihoods but inaccessible kinetically. Using 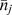 to signify the average value of species *j* due the dynamics of the system and 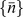 to indicate that all species are at their dynamical average values. Then the free difference between the average state that is accessible kinetically and the thermodynamically most probable state is,
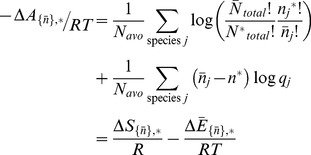
(19)


In a system in which there are no kinetic bottlenecks so that the kinetic and Boltzmann average states coincide, this free energy difference is 0. When the respective average states do not coincide, then Equation 19 represents the excess free energy production, the change in configurational entropy and heat dissipation/production that may be present in a non-equilibrium system, depending on whether the reactions occur on similar timescales.

Since the thermodynamically most probable path through a set of reactions is the also the path with the highest change in state entropy, the excess state entropy always positive – the system is moving away from the thermodynamically most probable state - and consequently always results in a decrease in the state entropy of the system. In other words, sampling according to Boltzmann likelihoods will always result in greater dissipation of state entropy than sampling according to kinetic likelihoods.

### Implementation and Simulation Procedure

The methods discussed above were implemented in a stochastic simulation using a Markov model. Changes of state in a stochastic simulation are based on probabilities rather than the likelihoods used in Equation 8. The relationship between probability 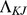 and likelihood 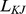 is 
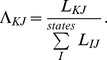



The sum over states 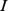 includes state 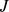 because there is a probability that there is no change in the state. In a Markov model the probability 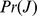 of state 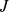 at step 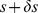 is then,
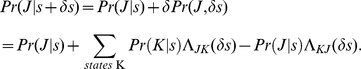



At each step 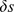 in the simulation, the counts of all species are updated according to the reaction that is chosen, which results in a new state. Consequently, new likelihoods and probabilities are determined based on the new configuration, and the process is repeated.

The molecular partition functions used in the likelihoods are calculated from standard free energies of formation of each molecular species. The partition functions are related to the chemical potential through the relationship [17],
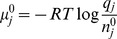
where 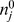 is the number of particles of species 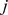 present at equilibrium. In the simulation, the total number of particles is allowed to vary using the statistical ensemble 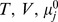 (grand canonical), where 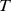 is the temperature and 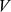 is the volume. In this ensemble, the simulation will adjust the number of particles for each chemical species in attempt to match the Boltzmann probability distribution for that species, subject to the boundary conditions of the non-equilibrium system.

The standard free energies are adjusted for ionic strength of the solvent using the Debye-Hückel Equation and for pH for ionizable species. In the results reported below, standard free energies of formation were obtained from the Equilibrator web server [22] and consisted of standard free energy values from both experiment [23], [24] and group contribution methods. Care was taken to ensure that standard free energy values were used consistently – experimental values were compared to only experimental values and group contribution values were compared only to other group contribution values.

## Results

### Application to the Tricarboxylic Acid Cycle

**Figure 7:**
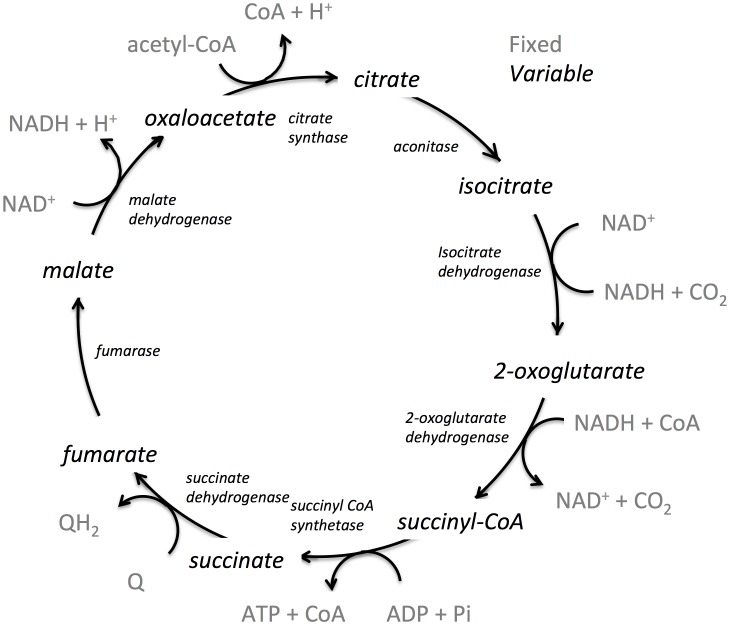
The tricarboxylic acid cycle of E. coli used in the simulations. Each intermediate in black is allowed to vary in concentration during the simulation, while each cofactor, CO_2_, and Acetyl CoA in grey are held constant.

To demonstrate the ability of state-based simulations to elucidate physical insights and principles in biochemical systems, the tricarboxylic acid cycle (TCA cycle) from Escherichia coli was simulated. The TCA cycle is possibly the most fundamental pathway in organisms [25]. Many variations exist in which the cycle is used to dissipate material and energy, store material and energy, and provide metabolic precursors for biosynthetic processes. The TCA cycle of *E. coli* (shown in Figure 7), *Saccharomyces cerevisiae* and mammals are very similar in structure, and hence are familiar to most researchers.

The *E. coli* TCA cycle takes as input acetyl-CoA, which is derived from pyruvate during glycolysis. After one turn of the cycle, the two carbons of the acetyl group are oxidized to two carbon dioxide molecules, with the energy derived from the oxidation being used to form one ATP from one ADP, three NADH from three NAD+, and the reduction of one electron carrier, typically a quinone. The overall reaction of the cycle is,
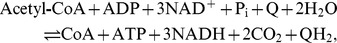
where 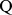 and 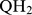 represent an oxidized and reduced electron carrier, respectively.

To demonstrate the thermodynamic concepts discussed above, simulations were carried out using the metabolite levels reported by Bennett, *et al*., [26] for exponential growth of *E. coli* under high levels of glucose. All cofactor levels were held fixed during the simulation including ATP, ADP, orthophosphate, NAD+, NADH, CoA, 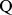 and 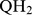, as well as the starting material, acetyl-CoA, and the final product, CO_2_. The levels of the reaction intermediates (citrate, isocitrate, 2-oxoglutarate, succinyl CoA, succinate, fumarate, malate and oxoaloacetate) were allowed to vary. The species that are fixed and variable are summarized in Figure 7.

The levels of the reaction intermediates are expected to change significantly from those reported by Bennett since a model of the TCA cycle in isolation is not representative of the full capability of *E. coli* metabolism. Consequently, the simulation was allowed to run for 100M steps, allowing the state entropy (Equation 14) to reach a stable value.

**Figure 8:**
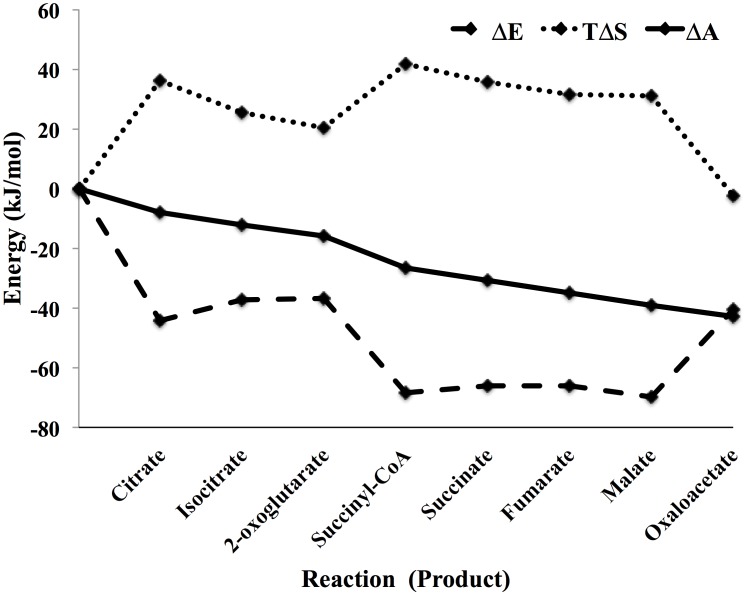
Energy (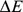), entropy (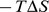) and free energy (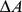) profiles for traversing the TCA cycle shown in Figure 7 using the data on metabolite levels from Bennett et al. Each reaction is labeled by the reaction product.

The free energy profile for traversing the TCA cycle is shown in Figure 8, along with the profile of the configurational entropy and the energy. Several general trends are apparent in the profile. First, as expected from the discussion above there is an energy-entropy compensation throughout the cycle. Each decrease in energy is associated with a decrease in configurational entropy (increase in 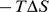) and vice versa. Also, as expected from the discussion above, the entropy change for going around the cycle is close to zero. The residual entropy for traversing the cycle is −2.36 kJ/mol, which is the entropy needed to bring the cofactors back to their original values after one turn of the cycle, after which the system would be precisely at a steady state. Consequently, the free energy decrease differs from the decrease in energy by 2.36 kJ/mol, also. The overall free energy change is −42.99 kJ/mol, consistent with operation of the cycle in the oxidative direction.

**Figure 9:**
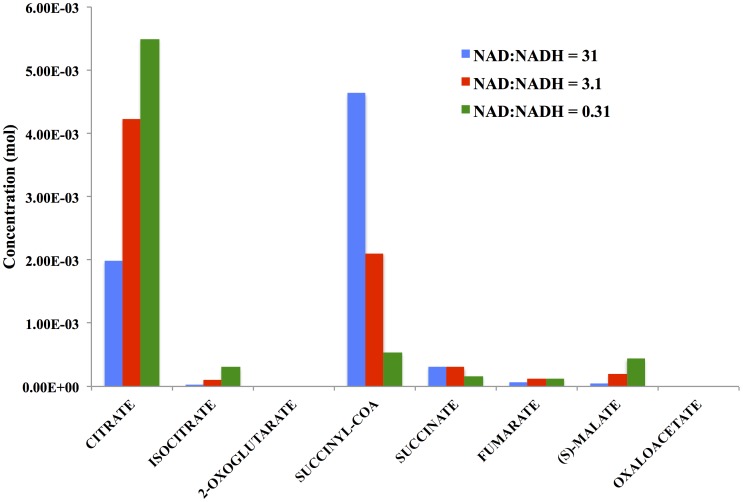
Predicted metabolite levels for each reaction intermediate shown in Figure 7 under conditions in which the NAD+:NADH ratio is 31 (Bennett et al. [26]), or decreased by either 0.1-fold or 0.01-fold. The compounds with the lowest mean concentrations have the highest variability. The coefficients of variation for 1000 simulation steps are (from high to low): oxaloacetate, 1.60e–02; 2-oxoglutarate, 1.66e–02; succinate, 1.10e–05; fumarate, 2.30e–05; isocitrate, 5.15e–06; malate, 5.20e–06; citrate, 2.73e–07; succinyl-CoA, 5.81e–08.

The free energy change for each reaction is nearly the same, in agreement with Equations 12 and 13. The free energies for the reactions for the conversion of oxaloacetate to citrate (−7.92 kJ/mol) and 2-oxoglutarate to succinyl CoA (−10.24 kJ/mol), differ somewhat from the mean of the other reactions (−4.12+/−0.16) due to the constraints imposed by the cofactor concentrations – that is, the boundary conditions of the simulation. Specifically, even when the reactants for these reactions, oxaloacetate and 2-oxoglutarate, are at the lowest possible concentration levels for which a free energy can be determined - one molecule per cell - the reaction free energies are relatively large because of the ratios of the cofactor reactant-product pairs [acetyl-CoA]/[CoA] and ([NAD][CoA])/([NADH][CO_2_]), respectively. The resulting steady-state levels of the metabolites are shown in Figure 9.

### Changes in Cofactor Levels alter Concentrations of Metabolites

Alteration of the cofactor levels results in changes in the concentrations of the cycle intermediates. Also, shown in Figure 9 are the concentration levels of the eight reaction intermediates when the ratio of NAD^+^:NADH decreases by 10- and 100-fold from the values reported by Bennett, et al. Since three of the reactions in the TCA cycle use NAD^+^ as a reactant and NADH as a product, a reasonable expectation would be that a decrease in the NAD^+^:NADH ratio decreases the thermodynamic driving force for the forward reaction and results in an increase in the other reactants of the respective reactions as well.

This is indeed the case. The sequential isocitrate dehydrogenase and 2-oxoglutarate dehydrogenase reactions both use NAD^+^ as a reactant and NADH as a product and for this purpose can be considered as a single reaction in which citrate is transformed to 2-oxoglutarate, using 2 NAD+ and producing 2 NADH. When the NAD+ concentration is lowered by 0.1- and 0.01-fold relative to NADH, the level of citrate increases by 2- and 3-fold, respectively, while the intermediate isocitrate increases by 4.5 and 12-fold, respectively.

Likewise, the malate dehydrogenase reaction converting malate to oxaloacetate also uses NAD^+^ as a reactant and produces NADH as a product. Decreasing the NAD^+^:NADH ratio by 0.1- and 0.01-fold results in an increase in the reactant malate by 4- and 10-fold, respectively. The results here are in line with chemical intuition. However, because the reactions are coupled, the affects of changing cofactor concentrations are not always predictable. In general, the results depend heavily on whether each reaction is more or less equally likely, as in this case, or whether one or a few reactions are further away from equilibrium than the others. In fact, increasing the overall driving force on a pathway does not guarantee a proportional increase in flux through the pathway. In other scenarios for the TCA cycle, decreasing the ATP concentration by 10-fold has a more significant affect on flux than decreasing the CO_2_ concentrations by an equal amount, even though the latter lowers the free energy of the pathway by twice as much. Because flux is an emergent property of the entire pathway, such affects are hard to predict without a simulation.

The use of simulations based on states and prediction of metabolite levels can clearly be a game changer for modeling applications in synthetic biology. Several efforts in over-producing target chemicals have focused on redirecting carbon flow, by knock-out of key genes in alternative pathways for example, but altering the thermodynamics of the target pathway – either by changing the redox state or other means – will likely prove to be more fruitful and, ultimately, necessary.

## Discussion

Unfortunately, it's not currently possible to obtain all the necessary rate constants to model a system with specific time dependence. Besides the fact that each ortholog of an enzyme will have different rate constants, the challenge of obtaining accurate rate constants is much harder than one might imagine. Kinetic parameters vary significantly with solution conditions – pH, ionic strength, dielectric, etc. While thermodynamic parameters also vary with solution conditions, the variation is significantly more predictable using modern computational chemistry methods [27], [28]. In fact, useful estimates of standard free energies of reaction can be obtained *en mass* for large scale modeling from resources such as the Thermodynamics of Enzyme-Catalyzed Reactions Database at NIST [23], the Biochemical Reactions Thermodynamics Database [29], and the eQuilibrator web server [22]. Given the variability of kinetic parameters due to physical influences and differences in rates between orthologs, it is debatable whether achieving a full-scale kinetic simulation is a reachable goal. Currently, flux-based models are the best that one could do for modeling large-scale processes in metabolism. Flux-based approaches are not based on law of mass action, so prediction of energy requirements and metabolite levels is difficult without assumptions regarding the relationship between flux and free energy changes. In this light, the development of metabolic models based on statistical thermodynamics simulations is a reasonable alternative.

Whether the use of statistical thermodynamics based on the standard chemical potential for simulating metabolism is an appropriate modeling choice depends on the question that one is trying to address. The assumption inherent in the use of the standard chemical potential is that each change of state occurs with a frequency proportional to the thermodynamic driving force for the respective reaction. A similar assumption is used in transition state theory – that the reactant species and the transition state species are distributed according to a Boltzmann distribution. This assumption is turned into a rate law in the latter case by multiplying the Boltzmann likelihood by the frequency of a bond vibration – the universal frequency factor. In the case of modeling metabolism, one does not necessarily need to model the time dependence of each reaction explicitly to gain insight into many emergent properties of entire pathways.

The use of simulations based on statistical thermodynamics is fundamentally a numerical search for a thermodynamically optimal path from reactants to products. In comparison to experimental measurement of absolute metabolite values or a precise kinetic simulation, the metabolite distributions will likely differ. However, these differences should be significantly less when evaluating relative changes in metabolite levels, and the principles and insight learned from the modeling exercise should nevertheless be the same. Moreover, the difference between experimentally measured metabolite levels and metabolite levels predicted from a simulation, whether based in kinetic rate laws or thermodynamics, will predominately depend on enzyme regulation, of which both simulation technologies are capable of including. Even if the system is not a high fidelity model of the time-dependence, the principles will be the same.

If one were to assume that the simulation represented an underlying kinetic model, then one would need to include in Equation 8 a coefficient to alleviate the assumption that each reaction occurs with a frequency proportional to the thermodynamic driving force on the reaction. Otherwise, the model will characterize a thermodynamically optimal process, rather than a specific system. However, this assumption may not be unreasonable for modeling metabolism. Biological systems are mutable and natural selection will favor those organisms that most effectively consume free energy [30]–[33], and the system in which each reaction occurs in proportion to the thermodynamic driving force on the reaction will be at the lowest absolute free energy. As suggested elsewhere, it is likely that the emergence of biological function does not depend on the precise values of the catalytic rates [34].
